# Comparison of *TCN-2* (776C>G) Gene Polymorphism and Vitamin B12 Status with Different Body Mass Index among Saudi Adults

**DOI:** 10.3390/life13051185

**Published:** 2023-05-15

**Authors:** Fauzia Ashfaq, Abeer M. Aljaadi, Afnan S. Salaka, Essra A. Noorwali, Fahmida Khatoon, Mohammad Idreesh Khan

**Affiliations:** 1 Department of Clinical Nutrition, College of Applied Medical Sciences, Jazan University, Jazan 45142, Saudi Arabia; 2 Clinical Nutrition Department, Faculty of Applied Medical Sciences, Umm Al-Qura University, Makkah 21955, Saudi Arabia; 3 Laboratory Medicine Department, Faculty of Applied Medical Sciences, Umm Al-Qura University, Makkah 21955, Saudi Arabia; 4 Department of Biochemistry, College of Medicine, University of Hail, Hail P.O. Box 2440, Saudi Arabia; 5 Department of Clinical Nutrition, College of Applied Health Sciences in Ar Rass, Qassim University, Ar Rass 51921, Saudi Arabia

**Keywords:** body mass index, *TCN-2* gene, polymorphism, obesity, vitamin B12

## Abstract

Background: Overweight and obesity (OO) are significant public health issues, and many elements, including genetics, epigenetics, sedentary lifestyle, comorbid conditions, psychological and environmental pressures, have been linked to OO. More than 2 billion people are presently impacted by the global obesity epidemic, which is still advancing relentlessly. It is a significant public health concern and a major contributor to healthcare costs, because it increases the chance of developing conditions such as heart disease, stroke, type 2 diabetes, and chronic kidney disease (CKD). Using the ranges of 18.5–25 for normality, 25–30 for overweight, and 30 for obesity, BMI (in kg/m^2^) is used to identify obesity. Vitamin deficiency is one of the causative factors associated with the increasing trend of obesity. Altered vitamin B12 status is a multifactorial trait; changes in B12 status are produced by several single nucleotide polymorphisms (SNPs) in various genes that interact with the environment. They also support coordinated efforts to alter the built environment that is causing the obesity pandemic. Therefore, the present study aimed to evaluate the *TCN-2* (776C>G) gene alteration and vitamin B12 levels with respect to different body mass index, as well as associating BMI with other biochemical parameters. Methods: 250 individuals were involved in the study; among them, 100 were in the healthy weight range category (BMI: 18.5 to <25 kg/m^2^), 100 were overweight (BMI: 25.0 to <30 kg/m^2^), and 50 were obese (BMI: >30 kg/m^2^). Participants visited during the screening program were subjected to blood pressure measurement, and further peripheral blood samples were drawn from all the participants in plain as well as in EDTA vials for biochemical (lipid profile and vitamin B12 level) analysis and single nucleotide polymorphism studies. Extracted DNA from whole blood collected in EDTA vials using kit protocol was used for genotyping by PCR-RFLP. Results: The levels of systolic (*p* < 0.0001) and diastolic blood pressures (*p* < 0.0001), HDL (*p* < 0.0001), LDL (*p* = 0.04), TG (*p* < 0.0001), cholesterol (*p* < 0.0001), and VLDL (*p* < 0.0001) showed significant differences between healthy controls, overweight, and obese groups. The healthy control *TCN-2* (776C>G) genotypes were compared with those of overweight and obese participants, and compared to the healthy controls it was observed that overweight (*p* = 0.01) and obese (*p* = 0.002) subjects had significant differences in *TCN-2* (776C>G) genotypes. For genotypes CG and GG, the odds ratio was 1.61 (0.87–2.95; *p* = 0.12), and 3.81 (1.47–9.88; *p* = 0.005) for overweight participants, respectively, and obese participants’ calculated odds ratios were 2.49 (1.16–5.36; *p* = 0.01) and 5.79 (1.93–17.35; *p* = 0.001), respectively. The relative risk for genotypes CG and GG, was 1.25 (0.93–1.68; *p* = 0.12), 2.17 (1.12–4.17; *p* = 0.02) for overweight participants, while the obese participants’ calculated relative risks were 1.31 (1.03–1.68; *p* = 0.01) and 2.02 (1.12–3.65; *p* = 0.001), respectively. Vitamin B12 levels were analyzed, and it was observed that a significant difference existed among overweight (305.5 pmol/L, *p* < 0.0001) and obese patients (229 pmol/L, *p* < 0.0001), respectively, as compared to healthy controls (385.5 pmol/L). Correlation analysis showed a significant association of vitamin B12 level with TG, cholesterol and VLDL; it showed a negative correlation, suggesting that decreases in B12 levels may impact the lipid profile. Conclusion: The study concluded that a predisposition to the GG genotype of *TCN-2* gene polymorphism (776C>G) may increase susceptibility to obesity and the related complications, and higher odds and relative risk for the GG genotype may increase the risk of having obesity and further related complications. Lower vitamin B12 levels were linked with obesity and overweight, and impaired lipid parameters suggested that lower vitamin B12 may impact the altered lipid profile.

## 1. Introduction

Obesity and being overweight (OO) are significant global public health issues. Many factors, including genetics, epigenetics, a lack of exercise, comorbid conditions, psychological stress, and environmental pressures, have been proven to contribute to OO [1]. There is mounting evidence that genetics play a role in the emergence of OO. Over the past 20 years, diabetes and cardiovascular diseases have dramatically increased, and obese and obesity have become the most expensive chronic diseases in the world [2,3]. Obesity is a condition of metabolic alteration; a person’s total energy intake is changed by their dietary composition, total energy expenditure, or both in order to disrupt their energy balance [4]. OO has been observed to be growing day by day in all ethnic groups [5]. Transcobalamin II is a protein coded by the *TCN-2* gene, which transports cobalamin to the cells or tissues [6]. Cobalamin, or vitamin B12, is a crucial water-soluble vitamin that must be consumed by people to preserve health, and vitamin B12 insufficiency has been associated with a variety of consequences, including a higher incidence of macrocytic anemia and neuropsychiatric symptoms [7], cardiovascular diseases [8], and the onset of different forms of cancer [9,10]. Transcobalamin (TC) transports vitamin B12 in the bloodstream and provides it to the cells [11]. After being bound by haptocorrin in the stomach, and intrinsic factor in the duodenum, vitamin B12 is transported to TC within the enterocyte and released into the blood. The vitamin B12-TC combination is then taken up by TC-R-mediated receptor-mediated endocytosis [11]. The *TCN-2* gene, which contains 9 exons and is found at 22 q11-13.1, spans an area of 18 kb [12].

At this time, genetic research on vitamin B12 status points to a multifactorial trait, wherein changed B12 status is caused by many single nucleotide polymorphisms (SNPs) in several genes interacting with the environment [13]. It has been reported that *TCN-2* is associated with waist circumference (WC) and its variant mediates obesity-related anthropometric traits [14].

Those who are overweight, including obese children and adolescents, are more likely to suffer from vitamin B12 insufficiency [15]; this also includes obese women with polycystic ovary syndrome [15] and obese pregnant women [16]. Changes in the TC protein could have an impact on how vitamin B12 binds to it, or how TC-R recognizes the vitamin B12-TC complex, which could have an impact on the cells’ ability to absorb vitamin B12. Therefore, it is important to understand the contention behind obesity, overweight, its association with the *TCN-2* (776C>G) (rs1801198) gene alteration, vitamin B12 levels, and its association with smoking among the Saudi population.

## 2. Methodology

### 2.1. Subject Selection

The present study included a total of 250 individuals; among them, 100 were in the healthy weight range category (BMI: 18.5 to <25 kg/m^2^), 100 were overweight (BMI: 25.0 to <30 kg/m^2^), and 50 were obese (BMI: >30 kg/m^2^). This study has been approved by the biomedical research ethics committee of Umm Al-Qura University, Makkah, Saudi Arabia (Approval No-HAPO-02-K-012-2022-01-923). Informed consent was obtained from all the participants who were invited for a screening of obesity, with a fasting condition, at the university clinic. Participants who had any other complications were excluded. Participants who visited the clinic were asked to rest for 5 min, and their blood pressure was recorded twice in the supine position to avoid any possible alteration. Their height and weight were recorded with an electronic Tanita measurement device. Most importantly, participants who had type 2 diabetes or any other disease or complications were excluded; we aimed only to study the single nucleotide polymorphisms and vitamin B12 status of the overweight and obese participants.

### 2.2. Subject Selection and Blood Sample Collection

After the screening, based on the inclusion criteria, participants were randomly included and categorized by BMI into the overweight or obesity groups, after patients with diseases such as diabetes or any chronic disease or malignancies had been excluded. Participants with a normal BMI (18.5 to <25 kg/m^2^) were considered healthy controls. Further, a total of 5.0 mL peripheral whole blood was drawn from each participant into two different vials; 3 mL was drawn into a plain vial, left to stand at room temperature for few minutes, and then further centrifuged at 3000 rpm for 10 min to separate the serum. It was stored at −80 °C for biochemical and vitamin B12 level assessment.

### 2.3. DNA Extraction

Blood samples were collected in EDTA vials from all the participants; they were subjected to DNA extraction for polymorphism studies, which was carried out using commercially available kits (Geneaid) and following the kit’s instructions. During the DNA extraction, cells were mixed with RBCs lysis buffer, and protinase was added before incubation at 55 °C. A further column was used, and subsequently, wash buffer was added.

Finally, the mixture was eluted with the elution buffer provided in the kit (https://www.geneaid.com/Genomic-DNA-Purification/GS, accessed on 15 November 2022).

### 2.4. Biochemical Parameters and B12 Level Assessment

Serum of all the participants stored at −80 °C was taken out, thawed and used for lipid profile and vitamin B12 analyses using an electrochemiluminescence immunoassay (Cobas e411, Roche, Basel, Switzerland). A vitamin B12 level < 148 pmol/L was considered deficient, and above this figure was considered a normal level [17].

### 2.5. TCN-2 Gene Alteration among Overweight, Obese and Healthy Subjects

**Figure 1:**
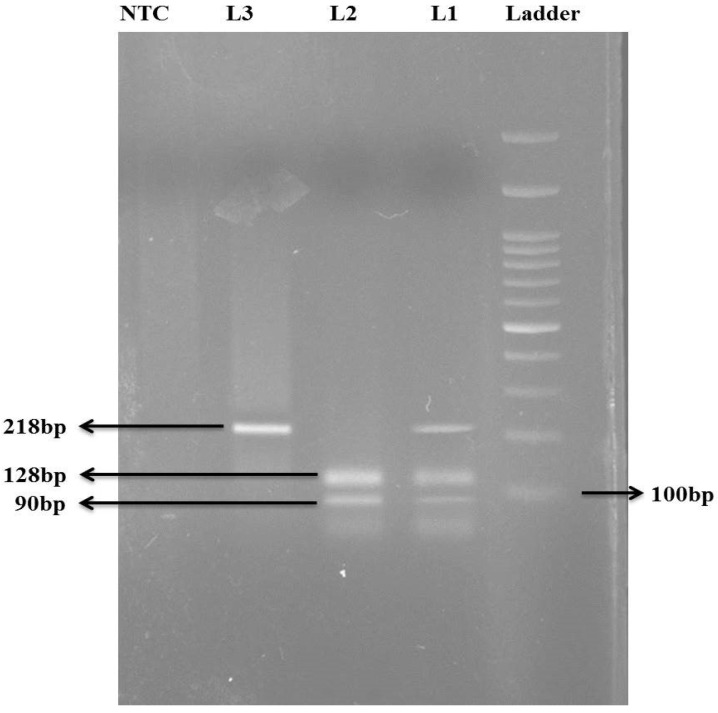
Gel picture of *TCN-2* (776C>G) polymorphism; ladder (100 bp), L1 (heterozygous CG), L2 (homozygous mutant GG), L3 (homozygous CC), NTC (non-template control).

The *TCN-2* (776C>G) single nucleotide polymorphism was determined with the PCR-RFLP method, using extracted DNA from all the participants. For the PCR reaction, after the quantification, 100 ng of DNA was used as a template, and 25 pmol of each primer was prepared, such as forward and reverse (0.25 μL), 10 mM dNTPs (2.5 μL), 20 mM MgCl_2_ (1.5 μL), and 5 U/μL Taq polymerase (0.3 μL) with 10× Taq Buffer (2.5 μL) containing master mix (Fermantas, Waltham, MA, USA). Nuclease-free water (14.7 μL) was added to make a total reaction volume of 25 μL. Initial denaturation took place at 94 °C for 10 min, followed by denaturation at 95 °C for 40 s, annealing at 64 °C for 40 s, and extension at 72 °C for 40 s for 40 cycles. The final step was extension at 72 °C for 10 min, under PCR cycling conditions. After the amplification, the product was visualized under a UV transilluminator to confirm the amplification. The primer (5′GTCAGGTGCTGGAACACCTAG3′) and the 2.0 μM reverse primer (5′CGTTCTGAACCACAAGACCTA3′) were used to amplify the target sequence, and were digested by the *Mva*I enzyme. Based on the digestion product, the wild-type CC genotype showed no digestion by the enzyme, and had 1 band of 218 bp. The heterozygous CG genotype had 3 bands of 218 bp, 128 bp and 90 bp, and the mutant homozygous GG genotype had 2 bands of 128 bp and 90 bp. Based on the digestion data, the participants were recorded in Excel as homozygous, heterozygous and mutant homozygous (Figure 1).

### 2.6. Statistical Analysis

All the statistical analysis was performed using GraphPad Prism 5.0, and the statistical program for social science (SPSS) version 20 was used to analyze all data. The genotyping frequencies between the cases and healthy control groups were compared using the Chi-square test, and values under 5 were examined using Fisher’s exact test. Allele frequency was calculated using the Hardy–Weinberg equilibrium (HWE) equation, and an odds ratio was used to estimate the relationship between the *TCN-2* gene and the risk of OO. Quantitative data were analyzed using a Kruskal–Wallis test followed by post hoc Dunn test to compare more than two groups, as the outcome is nonparametric. A correlation analysis was performed for vitamin B12 with other biochemical parameters to understand the link between vitamin B12 and the biochemical parameters. *p* values < 0.05 were considered statistically significant.

## 3. Results

### 3.1. Demographics of the Participants

The study included a total of 250 participants; among them, 100 were healthy controls free from any kind of disease and with no metabolic disorder, 100 were overweight, and 50 were obese, based on BMI (Table 1). Among the participants, of the healthy controls, 81% were males and 19% were females; the overweight category had 87 % males and 13% females, while among the obese category, 92% were males and 8% were females. The mean age of the healthy controls was 38.38 ± 6.65, of the overweight group was 37.61 ± 6.74, and of the obese group was 39.24 ± 6.69.

### 3.2. Comparison of Biochemical Parameters between Healthy Controls, Overweight and Obese Participants

Biochemical parameters were analyzed and compared between the control, overweight and obese categories (Table 2). It was observed that the systolic blood pressure (*p* < 0.0001), diastolic blood pressure (*p* < 0.0001), HDL (*p* < 0.0001), LDL (*p* = 0.04), TG (*p* < 0.0001), cholesterol (*p* < 0.0001), VLDL (*p* < 0.0001) showed significant differences between control, overweight and obese groups. The average systolic blood pressure of the healthy control participants was 126.6 ± 7.0, of overweight participants was 148.1 ± 13.69, and of obese participants was 150.7 ± 17.80. The average diastolic blood pressure was 85.58 ± 6.34, 96.20 ± 7.58 and 97.06 ± 7.86 among the healthy controls, overweight and obese participants, respectively. The HDL was higher in healthy controls (45.05 ± 13.20), while comparatively low in the overweight (39.22 ± 11.10) and obese (34.86 ± 7.13) groups. Higher TG was observed in overweight (216.2 ± 32.44) and obese (222.7 ± 43.50) participants compared to healthy controls (165.6 ± 18.29). The level of cholesterol was also high in overweight (233.9 ± 25.50) and obese (248.8 ± 7.59) participants compared to healthy controls (186.9 ± 35.20), as well as higher VLDL among overweight (32.26 ± 4.58) and obese (36.78 ± 4.59) participants compared to healthy controls (22.97 ± 4.28).

### 3.3. Genotype Distribution among Healthy Controls, Overweight and Obese Participants

We observed significant differences in *TCN-2* (C to G) genotypes among controls and overweight (*p* = 0.01) and obese (*p* = 0.002) groups (Table 3). The wild-types CC genotype frequencies in the control, overweight and obese groups were 59%, 42%, and 32%, respectively, the heterozygous CG genotype frequency was 34%, 39%, and 46%, respectively, and the mutant GG genotype frequency was 7%, 19% and 22%, respectively. The Hardy–Weinberg equilibrium equation was used to calculate the allele frequency; the calculated allele frequency of C and G in the control group was 0.76 and 0.24, respectively. For the overweight group, it was 0.60 and 0.40, respectively, and in the obese group, it was 0.55 and 0.45, respectively. Higher allele frequency was observed for overweight and obese participants compared to the controls.

### 3.4. Odds Ratio with Respect to TCN-2 Genotypes in Healthy Controls, Overweight and Obese Participants

By using wild-type genotypes as a reference, odds ratios for participants who were overweight or obese were determined (Table 4). For genotypes CG and GG, the odds ratios were 1.61 (0.87–2.95) and 3.81 (1.47–9.88), respectively. With regard to the CG and GG genotypes, the odds ratios for obese patients were 2.49 (1.16–5.36) and 5.79, respectively (1.93–17.35).

### 3.5. Relative Risk with Respect to TCN-2 Genotypes in Healthy Control, Overweight and Obese Participants

For those who were overweight or obese, relative risk was estimated using wild-type genotypes as a baseline (Table 5). For genotypes CG and GG, the odds ratios were 1.25 (0.93–1.68) and 2.17, respectively (1.12–4.17). Among those who were obese, the odds ratios for the CG and GG genotypes were 1.31 (1.03–1.68) and 2.02, respectively (1.12–3.65).

### 3.6. Comparison of Vitamin B12 Levels between Healthy Control, Overweight and Obese Participants

**Figure 2:**
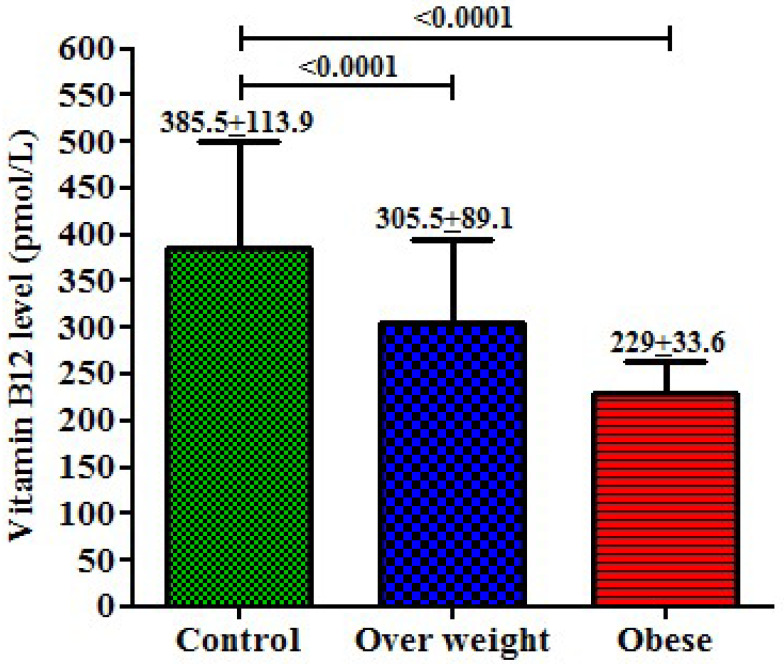
Levels of vitamin B12 and comparisons between healthy controls, overweight and obese participants.

Levels of vitamin B12 were assayed and compared between healthy controls, overweight, and obese participants, and a significant difference was observed among them (Figure 2). The healthy control participants had an average 385.5 pmol/L vitamin B12 level, overweight participants had an average 305.5 pmol/L (*p* < 0.0001), and obese participants had an average 229 pmol/L (*p* < 0.0001) vitamin B12 level. Comparatively, we observed lower vitamin B12 levels among the overweight and obese participants compared to the healthy controls; however, all the measurements fell within a normal range.

**Figure 3:**
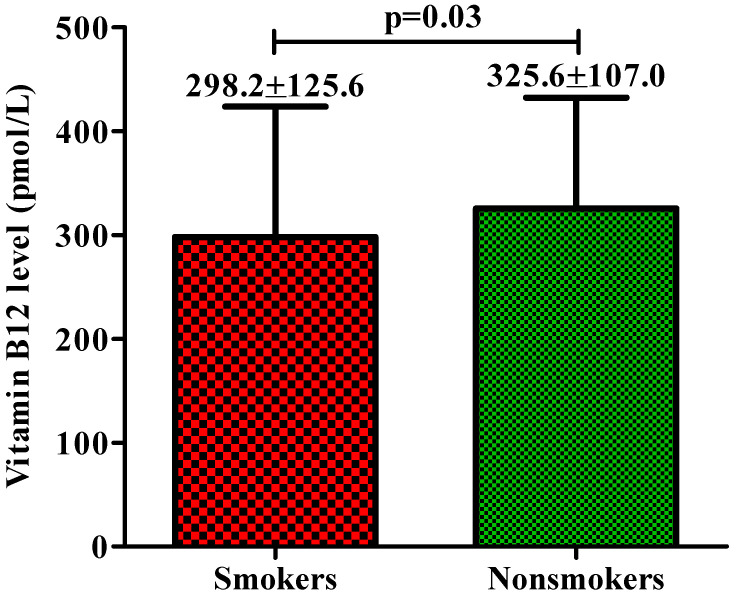
Comparison of vitamin B12 levels between smokers and nonsmokers.

Association of vitamin B12 level with smoking status: This study included 12.4% smokers and 87.6% were nonsmokers and compared vitamin B12 level among them (Figure 3). A significant difference in vitamin B12 levels was observed between smokers and nonsmokers; smokers had an average vitamin B12 level of 298.2 mol/L, while nonsmokers had an average vitamin B12 level of 325.6 pmol/L, suggesting smoking could have a negative impact on vitamin B12 level; however, deficiency is not necessarily present in smokers.

### 3.7. Association Vitamin B12 Level with Blood Pressure, Biochemical Parameters

**Figure 4:**
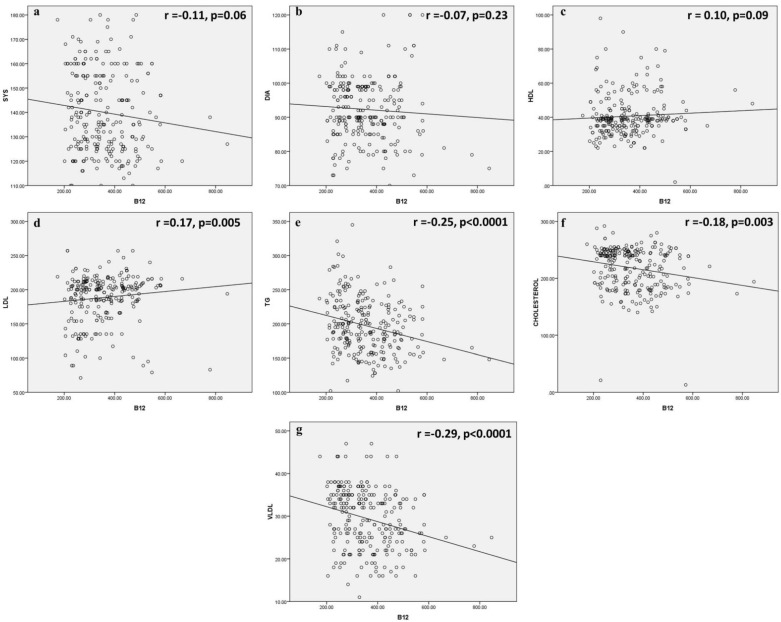
Correlation analysis of vitamin B12 levels with different parameters (**a**) systolic blood pressure, (**b**) diastolic blood pressure, (**c**) HDL, (**d**) LDL, (**e**) TG, (**f**) cholesterol and (**g**) VLDL.

To understand the link between vitamin B12 and blood pressure (both systolic and diastolic), HDL, LDL, TG, cholesterol, and VLDL level, a correlation analysis was performed to estimate (Figure 4) their interconnection. It was observed that the level of vitamin B12 was associated with LDL (*p* = 0.005), TG (*p* < 0.0001), cholesterol (*p* = 0.003), and VLDL (*p* < 0.0001). The analysis showed that an increase in B12 level may correlate with a decrease in TG, cholesterol and VLDL.

## 4. Discussion

The protein transcobalamin binds to vitamin B12 and carries it from the ileum to the tissues [18]. Transcobalamin protein Pro259Arg substitution is caused by *TCN-2* (776C>G) single nucleotide polymorphism [19]. The availability of vitamin B12 to the tissues may be impacted by this polymorphism, which alters the binding of vitamin B12 by transcobalamin and is linked to a reduced concentration of holotranscobalamin (vitamin B12-bound transcobalamin) in plasma [20]. The *TCN-2* gene polymorphism (776C>G) was examined in the current study, and substantial changes in genotype distribution were found. Those who were overweight or obese (45%) were more likely to have the homozygous mutant genotype. The *TCN-2* gene polymorphism (776C/Gminor)’s G allele frequency was found in Brazilian (36%) [21], Latino (35%) [22], Nordic (44%) [23], Northern Irish (45%) [24], and Portuguese (48%) people. The odds ratio was calculated, and higher odds were observed due to the presence of heterozygous CG and homozygous mutant GG genotypes among overweight and obese participants.

Lower levels of vitamin B12 were observed to be linked with overweight and obesity compared to healthy controls. In a Portuguese cohort of 122 people, it was found that holo-transcobalamin concentrations were substantially correlated with the SNP rs1801198, and that G allele carriers had lower levels of holo-TC than C variant participants [25]. The 776GG homozygous variant encodes a protein that has a lesser affinity for binding vitamin B12 than the wildtype “C” allele [22]. Additionally, investigations have shown that polymorphisms in the TC protein inhibit the TC-R from recognizing the vitamin B12-TC complex, or diminish the binding of vitamin B12 to TC [26]. It has been asserted that the growth in obesity is a global problem which will have serious reproductive health consequences. Obesity increases the chance of experiencing a difficult pregnancy, which then elevates the possibility of the child developing childhood obesity and other non-communicable diseases as an adult [27]. It has also been suggested that vitamin B12 may affect adipogenesis [28,29]. Many epidemiological and observational studies have shown a connection between high BMI and low B12 levels [30], raising the level of glucose intolerance [31] and the risk of type 2 diabetes due to an unfavorable lipid profile [32]. Obesity has been connected to low B12 levels during pregnancy in both South Asian [33] and Caucasian [30] populations, and low B12 levels have been associated with increased BMI in kids and teenagers [34]. Furthermore, low B12 is more associated with obese youngsters than with healthy ones, and B12 levels are negatively correlated with the severity of obesity [34].

Surprisingly, a recent study in the Danish population revealed that higher BMI was strongly associated with decreased serum vitamin B12 concentrations [35]. Geographical regions of the Arab world are more likely to be obese than other regions, and genetic factors are thought to play a significant part in predisposing some Arab people to obesity [36]. According to a study by Al Batayneh KM et al. published in 2020, there is a substantial correlation between vitamin B12 insufficiency and the homologous variant of the *TCN-2* gene (776GG), and heterozygous people have an increased likelihood of developing the condition (OR = 5.6, 95% CI = 2.95 to 10.63) [37]. Nongmaithem SS et al. observed that variants in the B12-binding protein gene *TCN-2* have also been linked to circulating B12 levels [38], and that vitamin B12 bioavailability is controlled by the TCN-2 gene [39]. Alteration in TCN-2 is a mechanism that contributes to the pathogenesis of transcolabamin deficiency, and currently, many cases of transcolabamin deficiency have been reported worldwide [40], with only one case from China [41]. According to existing research, the existence of a *TCN-2* (CG + GG) variation has been linked to an increased chance of developing Crohn’s disease [42]. Those with *TCN-2* (GG) genotypes were found to be more susceptible to peripheral nerve impairment. [43]. It has been also shown that the G allele, CG, and GG genotypes all enhance the risk of oral cancer [44]. Patients with the GG genotype had considerably lower levels of holotranscobalamin, and the G allele of the protein that the *TCN-2* gene encodes is linked to less efficient transport of vitamin B12 from the blood to tissues [45].

It has been proposed that smoking may alter appetite and can influence nutritional intake and serum vitamin levels; the harmful contents of tobacco smoke, such as organic nitrites, nitro oxide, cyanides, and isocyanides in tobacco smoke may interfere with the metabolism of vitamin B12 [46]. According to a previous study, the serum levels of total vitamin B12 may not change between smokers and non-smokers, but smokers were less able to utilize this vitamin due to lower serum levels of its active form [46]. In 2003, Mannino DM et al. proposed that smoking exposure had a negative correlation with vitamin B12 levels [47]. Pregnant women who smoke and those who do not have been shown to have significantly different vitamin B12 levels, with smoking-exposed women having lower amounts of the vitamin [48]. In the same way, we also observed lower levels among smokers, but not decreased levels, compared to non-smokers; however, a continuous smoking habit may cause deficiency in the longer term. We observed a weak correlation of vitamin B12 with TG, cholesterol and VLDL, which can lead to altered levels of TG, cholesterol and VLDL.

## 5. Conclusions

Nowadays, overweight and obesity are major problems caused by both our lifestyle and our technologically advanced world, causing other metabolism-related diseases in society and bringing with them an economic burden. Genetic alteration in genes related to metabolism has been linked with poor metabolism, obesity and related complications. This study revealed that the *TCN-2* gene polymorphism (776C>G) increases susceptibility to obesity and may lead to other related complications or metabolism-related disorders. Higher odds and relative risk of having the GG genotype may increase the risk of having obesity. Lower vitamin B12 levels have been linked with obesity and overweight, and are also linked with impaired blood pressure and lipid parameters. A linear regression analysis showed a significant association of vitamin B12 levels with blood pressure and lipid parameters, suggesting that alterations in B12 levels may affect TG, cholesterol and VLDL levels. It is important for clinicians and obesity researchers to understand the impact of vitamin B12 deficiency leading to alterations in lipid parameters in obese individuals. Understanding these disturbances can help us to address the risk factors contributing to obesity in the Saudi population, for better management of the condition; with better management, we may overcome the problem of obesity and its associated complications.

## Figures and Tables

**Table 1:** Baseline characteristics of study participants.

Parameter		Healthy Controls (*n* = 100)	Overweight (*n* = 100)	Obese (*n* = 50)
Gender	Male	81 (81%)	87 (87%)	42 (92%)
	Female	19 (19%)	13 (13%)	8 (8%)
Age (years)		38.38 ± 6.65	37.61 ± 6.74	39.24 ± 6.69

**Table 2:** Biochemical parameter comparison among controls, overweight and obese participants.

Biochemical Parameters	Healthy Controls (*n* = 100)	Overweight (*n* = 100)	Obese (*n* = 50)	*p* Value *
Systolic blood pressure	126.6 ± 7.0	148.1 ± 13.6	150.7 ± 17.8	<0.0001 *
Diastolic blood pressure	85.5 ± 6.3	96.2 ± 7.5	97.0 ± 7.8	<0.0001 *
HDL-c (mg/dL)	45.0 ± 13.2	39.2 ± 11.1	34.8 ± 7.1	<0.0001 *
LDL-c (mg/dL)	181.6 ± 40.1	196.9 ± 28.6	186.6 ± 30.7	0.04 *
TG (mg/dL)	165.6 ± 18.2	216.2 ± 32.4	222.7 ± 43.5	<0.0001 *
Cholesterol (mg/dL)	186.9 ± 35.2	233.9 ± 25.5	248.8 ± 7.5	<0.0001 *
VLDL-c (mg/dL)	22.9 ± 4.2	32.2 ± 4.5	36.78 ± 4.5	<0.0001 *

HDL (high-density lipoprotein); LDL (low-density lipoprotein); TG (triglyceride); VLDL (very low-density lipoprotein) * is the *p* value indicator of significant.

**Table 3:** Genotype distribution and allele frequencies of the *TCN-2* gene among healthy controls, overweight and obese participants.

Variable		CC (%)	CG (%)	GG (%)	*p* Value	Allelic Frequency	
						C	G
TCN-2	Control (*n* = 100)	59 (59%)	34 (34%)	7 (7%)	-	0.76	0.24
	Overweight (*n* = 100)	42 (42%)	39 (39%)	19 (19%)	0.01	0.60	0.40
	Obese (*n* = 50)	16 (32%)	23 (46%)	11 (22%)	0.002	0.55	0.45

**Table 4:** Calculation of odds ratio with respect to *TCN-2* CC, CG, GG genotypes among overweight and obese participants.

**Gene**	**Genotype**	**Healthy Control**	**Overweight (n = 100)**	**OR (95%)**
TCN-2	CC (Pro/Pro)	59 (59%)	42 (42%)	Ref
	CG (Pro/Arg)	34 (34%)	39 (39%)	1.61 (0.87–2.95) (*p* = 0.12)
	GG (Arg/Arg)	7 (7%)	19 (5%)	3.81 (1.47–9.88) (*p* = 0.005)
**Gene**	**Genotype**	**Healthy Control**	**Obese (n = 50)**	**OR (95%)**
TCN-2	CC (Pro/Pro)	59 (59%)	16 (32%)	Ref
	CG (Pro/Arg)	34 (34%)	23 (46%)	2.49 (1.16–5.36) (*p* = 0.01)
	GG (Arg/Arg)	7 (7%)	11 (22%)	5.79 (1.93–17.35) (*p* = 0.001)

**Table 5:** Calculation of relative risk with respect to *TCN-2* CC, CG, GG genotypes among overweight and obese participants.

**Gene**	**Genotype**	**Healthy Control**	**Overweight (*n* = 100)**	**Relative Risk (95%)**
TCN-2	CC (Pro/Pro)	59 (59%)	42 (42%)	Ref
	CG (Pro/Arg)	34 (34%)	39 (39%)	1.25 (0.93–1.68) (*p* = 0.13)
	GG (Arg/Arg)	7 (7%)	19 (5%)	2.17 (1.12–4.17) (*p* = 0.02)
**Gene**	**Genotype**	**Healthy Control**	**Obese (*n* = 50)**	**Relative Risk (95%)**
TCN-2	CC (Pro/Pro)	59 (59%)	16 (32%)	Ref
	CG (Pro/Arg)	34 (34%)	23 (46%)	1.31 (1.03–1.68) (*p* = 0.02)
	GG (Arg/Arg)	7 (7%)	11 (22%)	2.02 (1.12–3.65) (*p* = 0.01)

## Data Availability

We confirm that the research data will not be shared in any public sphere or platform. The corresponding author can provide you with the datasets that were used and/or analyzed during this study.
